# Influence of Tooth Factors and Procedural Errors on the Incidence and Severity of Post-Endodontic Pain: A Prospective Clinical Study

**DOI:** 10.3390/dj8030073

**Published:** 2020-07-07

**Authors:** Lavanya Bamini, Anand Sherwood, Ana Arias, Savadamoorthi Kamatchi Subramani, Puridi Bhargavi

**Affiliations:** 1 Department of Conservative Dentistry and Endodontics, CSI College of Dental Sciences and Research, Madurai, Tamil Nadu 625 001, India; lavanyabamini@gmail.com (L.B.); anand.sherwood@gmail.com (A.S.); dr.bharu13@gmail.com (P.B.); 2 Department of Conservative and Prosthetic Dentistry, School of Dentistry, Complutense University, 28040 Madrid, Spain; 3 Division of Operative Dentistry, College of Dentistry, Jazan University, Jazan 114, Saudi Arabia; ksavadamoorthi@jazanu.edu.sa

**Keywords:** hot tooth, postendodontic pain, postoperative pain, preoperative pain, procedural errors

## Abstract

The objective of this prospective study was to assess tooth-related factors that play a role in the incidence of postoperative pain (PP) and determining if procedural errors influence PP occurrence. A total of 442 patients referred for root canal treatment met the inclusion criteria and were included in this prospective study. The same protocol was used in all root canal treatments. Patient, tooth, treatment related factors and the occurrence of procedural errors were registered. Incidence and intensity of PP was assessed at 24 and 48 h by telephonic interview and in person seven and 15 days after treatment. A logistic and ordinal regression analysis was used to assess the role of patient, tooth and treatment related factors in the incidence and intensity of PP, respectively. Preoperative and intraoperative factors differently affected the incidence of PP at the different time intervals. The presence of procedural errors did not significantly influence PP occurrence. The presence of preoperative pain and the need of additional anesthesia during treatment were associated with higher incidence of PP 24 and 48 h after treatment; the extent of apical enlargement played a significant role in the presence of PP after seven days of treatment; and the excessive occlusal load induced by the absence of a contralateral tooth was the only factor related to the maintenance of PP up to 15 days. In conclusion, the presence of preoperative pain, the need of additional anesthesia during treatment, the extent of apical enlargement and the excessive occlusal load induced by the absence of a contralateral tooth were related to a higher incidence of PP.

## 1. Introduction

Postoperative pain (PP) following root canal treatment is a disconcerting event for both clinicians and patients. A thorough understanding of the factors having a significant correlation with the presence of PP in Endodontics will allow the prediction of the event and warning patients about what to expect [1,2]. A Cochrane review report showed that incidence of PP is similar in both single and multiple-visit root canal treatment [3]. Postoperative pain after a root canal treatment can be either immediate or persistent long term [4,5]. Endodontic literature has reported incidence of PP varying from 3% to 65% 24 h after root canal treatment, that gradually decreased from 48 h onwards [5,6,7,8,9]. Such huge variations in results from different studies are likely due to the disparity in methods and procedures [7], selection of patients or differences in the expertise of clinicians [9]. PP is a complex multifactorial process influenced by factors inherent to patients, to the tooth to be treated and to the skills and decisions of the operator [9]. Some studies have reported that variables related to patient demographics such as age and gender may influence the presence of PP [8,9]; however, most of the factors that has been associated with a higher incidence of post-obturation pain in single-visit root canal treatment are tooth dependent. Among them the presence of preoperative pain, occlusal contacts, the type of tooth that has been treated, as well as, the existence of previous emergency endodontic treatments have shown to have significant association with PP [2,8,9].

The incidence of PP after a single or multiple-visit root canal treatment [8,9,10,11,12,13] is very well documented in the literature. However, the selection for single- or multiple-visit protocols is diverse. Some studies used randomization to choose the teeth to be treated with a single- or multiple-visit protocol, others include single-visit root canal treatment for both vital and non-vital teeth, and even some use multiple-visit root canal treatment with intra- canal medicament for all cases no matter if the tooth is vital or non-vital [10,11,12,13]. Clarity with regarding the decision-making process that leads to some procedural decisions is scarce and may have a role in the incidence or severity of PP. In fact, most clinicians make the decision for treating the tooth in a single or multiple-visit intraoperatively in a real clinical situation. Another relevant intraoperative decision is the extent of apical preparations. There is no agreement on the literature in the optimal apical enlargement for specific teeth. On the one hand, large apical preparation sizes may enhance the ability of irrigants to reach the apical portion and hence better reduce bacterial load; on the other hand, small apical preparations may reduce the incidence of preparation errors while helping to maintain the strength of the tooth by limiting dentin removal [14,15]. However, the extent of the apical enlargement performed by clinicians and the presence of preparation errors during root canal treatment procedures could also have an influence in PP and there is scarce information reported in the literature regarding their role in the incidence and severity of PP [1,6,9].

Therefore, the objective of this prospective observational study is to understand those tooth-related factors that play a role in the incidence of postoperative pain (PP) while determining if intraoperative circumstances (such as the extent of apical enlargement or the presence of procedural errors during access preparation, shaping and obturation of the root canal system) influence PP occurrence.

## 2. Materials and Methods

This prospective study was conducted after obtaining approval from Institutional ethics committee CSI College of Dental Sciences, Madurai, on 29 October 2014 (Protocol code: 0001/DE/2014) and informed consent from individual patients before treatment procedure. The STROBE checklist was followed.

Sample size calculation was performed using the information derived from a preliminary trial comparing the incidence of PP after 24 h in patients undergoing root canal treatments with registered absence or presence of procedural errors during treatment. These data estimated that anticipating a drop-out rate of 5%, a minimum sample size of 53 individuals with procedural errors and 212 with no procedural errors was required in order to detect differences for an effect size of 0.80 with an alpha error of 0.05.

Using convenience sampling, a total of 500 patients with pulpal and/or periapical symptoms due to caries who were referred for root canal treatment to the department of conservative dentistry and endodontics were included in this study. Periapical radiographs were taken to confirm the pulpal involvement of caries progression. A total of 8 operators with similar level of expertise carried out all treatments. When procedural errors were detected in 54 individuals, no more patients were recruited for the study.

Prior to starting the root canal treatments, the following data were registered:

- Preoperative medical history regarding presence of any systemic ailments;
- Gender and age of patient;
- Preoperative medication intake in the past 24 h;
- Presence of preoperative pain in the past 24 h in the concerned tooth;
- History of duration and intensity of previous pain in the concerned tooth;
- Pulp status assessed through thermal stimulation with ethyl chloride spray (Endo Frost, Roeko, Germany);
- Presence or absence of periapical radiolucency in the preoperative radiograph; PAI score was used to categorize the periapical appearance in the radiograph;
- Location of caries in the concerned tooth;
- Periodontal status of the concerned tooth;
- Presence of absence of contralateral tooth;
- Previous history of root canal treatment and pain experience after treatment.

If any data from the previous list was uncertain, the patient was excluded from the study.

Patients fulfilling the following criteria were also excluded from the study:

- Tooth presenting any sign of cracks;
- Tooth presenting mobility both in horizontal and vertical axis;
- Root canal retreatment;
- Pregnant women;
- History of medication for chronic pain or with a compromise immune response;
- Failure to obtain authorization for being part of the study.

All patients included in the study signed the informed consent and accepted to attend to recall visits. A total of 442 patients met the inclusion criteria and were included in the study.

All patients were anesthetized with 2.5 mL of 2% lignocaine local anesthesia containing 1:80,000 adrenaline (2% Lignox, Warren Pharmaceuticals, Mumbai, India). Inferior alveolar nerve block was administered for mandibular molar and premolar teeth and buccal and lingual/palatal local infiltration was used for maxillary teeth and mandibular incisors. After absolute rubber dam isolation, access cavity preparation was performed with a round diamond bur under water coolant. Patients with pulpal bleeding (from all canal orifices in multi rooted teeth) who also responded positively to pulp sensibility test were categorized as vital pulp and root canal treatment completed in a single visit. Absence of pulpal bleeding (from any canal orifices in multi rooted teeth) and absence or subdued pulp sensibility response was allotted as non-vital pulp with root canal treatment completed in multiple visits. In these cases, calcium hydroxide cement (Prime Dental, Bhiwandi, India) mixed with saline (NS 500 mL, sodium chloride 0.9%, Fresenius Kabi, India Pvt., Ltd, Pune, India) was used as intra-canal medicament and placed inside the root canal by coating the walls of the root canal using a K-file (Mani Co., Utsunomiya, Japan).

In order to standardize the endodontic treatment, the same standard evidence-based protocol was used in terms of shaping, cleaning and filling of the root canal system for all cases included in the study. In detail, a solution containing EDTA (15%) and carbamide peroxide (10%) (EndoPrep RC, Anabond Stedman Pharmaceuticals, Chennai, India) was used as canal lubricant during root canal negotiation. Working length was estimated using an apex locator (Root ZX mini, J Morita, Saitama, Japan) and confirmed using a periapical radiograph. Root canals were shaped using RaCe nickel–titanium (NiTi) rotary instruments (FKG, Dentaire, La Chaux-de-Fonds, Switzerland) and the endomotor EndoMate DT (NSK, Inc., Osaki, Japan) up to an apical size of either #20 or #25% and 6% taper. Further apical enlargement was performed with hand instruments up to sizes varying from #30 to #60 depending on the initial apical size of the root canal. Irrigation was performed with 3% sodium hypochlorite using a 23-gauge beveled needle and standard syringe. Chemo-mechanical preparation of the root canal system finished with manual dynamic activation of NaOCl. A lateral compaction technique was used for obturation. Zinc oxide eugenol-based sealer (DPI, Hyderabad, India) was placed twice into the canal using the master cone (having the same size as the apical file and 0.02 taper) as a carrier. Lateral compaction of size 15 gutta-percha cones (Diadent, Cheongju-si, Korea) with size 20-finger nickel–titanium spreaders was performed. A temporary restoration was placed, and occlusion was checked and adjusted when needed.

The following data were collected during the operative procedure:

- Hot tooth requiring additional anesthesia or patient experiencing pain while undergoing root canal treatment;
- Occurrence of any type of mishaps during access preparation, shaping or obturation of the root canal system;
- Final apical size.

All patients in this study were informed about possible pain experiences in the days after treatment and analgesics (aceclofenac 100 mg or paracetamol 500 mg) were prescribed if needed.

Incidence and intensity of PP was assessed 24 h, 48 h, 7 days and 15 days after root canal treatment. A trained assistant telephoned participants 24 h and 48 h after treatment and inquired about the incidence (yes/no) and intensity of PP. After 7 and 15 days, patients were recalled for clinical examination and PP was assessed by personal interview with the patient. The following simple verbal categorization defined by the need for and relief from an analgesic as recommended in a Cochrane review [3] was used to determine the incidence and intensity of pre- and postoperative pain:

- No pain;
- Mild pain: any discomfort that does not require analgesics;
- Moderate pain: persistent pain that requires and is relieved analgesics;
- Intense pain: persistent pain that is not relieved with analgesics.

Statistical analyses:

A multivariate statistical analysis was used to control the simultaneous relationships of the various factors that may intervene in the complex multifactorial outcome and to control any possible confounding factor. A logistic and an ordinal regression analysis were performed to assess the factors influencing the incidence and intensity of postoperative pain, respectively. A stepwise protocol was used to statistically enter and exclude factors from the regression model for a better global fitting. Apart from the size of apical enlargement and the presence of procedural errors during treatment, the multiple patient- and tooth-related factors collected pre- and intraoperatively were also introduced into the analysis to control any possible confounding factor. Odds ratios (OR) and their 95% CI were also estimated to measure the magnitude of the effect and quantify the strength of the association of the factor with occurrence of the event.

## 3. Results

### 3.1. Demographic Data

Out of the 442 that were included in the study, 183 (41.4%) were males and 259 (58.6%) females. Age distribution varied from 15 to 80 years (37.9% were younger than 30; 48.7% between 30 and 50; 13.4% older than 50). From the total, 332 patients received a single-visit root canal treatment in a vital tooth and 110 patients multiple-visit root canal treatment in a necrotic tooth. A total of 12.2% procedural errors were detected during treatment. Procedural errors occurred during access preparation, shaping and obturation of the root canal system. More specifically, the following errors occurred: perforations during access preparation (0.7%), instrumentation beyond apex (1.8%), root canal perforations (0.2%), instrument separation (2.9%), ledge formation (0.7%), canal obliteration (0.2%), master cone showing short or beyond apex on radiograph (1.8%), final obturation short or beyond apex (0.8%) and a combination of two or more of the above-mentioned errors (0.2%).

### 3.2. Incidence of PP

Table 1 shows the results for the incidence of postoperative pain and data distribution for relevant factors at the different time intervals. Of the 442 patients, seven patients did not respond satisfactorily to the telephone interview or attended the 7 and 15-day recalls.

Out of the 435 patients who correctly responded to postoperative pain assessment and attended postoperative visits, 128 patients (29.4%) experienced post treatment pain at any time interval: 119 patients (27.4%), 75 patients (17.2%), 16 patients (3.7%) and 3 patients (0.7%) reported, respectively postoperative pain 24 h, 48 h, 7 days and 15 days after treatment. Unscheduled interventions (flare ups) were needed in 5 patients (1.1%).

Preoperative and intraoperative factors differently affected the incidence of PP at the different time intervals. Gender, group of teeth, pulpal vitality status, the presence of periapical radiolucencies and the presence of procedural errors did not significantly influence PP occurrence after treatment. PP occurrence 24 and 48 h after treatment was significantly affected by the presence of immediate preoperative pain and the need of additional anesthesia (*p* < 0.05). Patients presenting for treatment with preoperative pain experienced a significantly higher incidence of PP with an OR = 3.2 (95% CI 1.9–5.2) and OR = 4.9 (95% CI 2.5–9.5), respectively 24 and 48 h after treatment. Moreover, if the treatment was performed in a hot tooth requiring additional anesthesia during treatment the OR for PP increased to OR = 4.8 (95% CI 2.5–9.1) and OR = 5.8 (95% CI 3–11.5), respectively 24 and 48 h after treatment. Both the presence of immediate preoperative pain (*p* = 0.004) and further apical enlargement (*p* = 0.022) played a significant role in the presence of PP after 7 days of treatment. Patients with preoperative pain experienced a significantly higher incidence of PP with an OR = 6.6 (95% CI 1.8–23.6) and further apical enlargement significantly increased the incidence of PP with an OR = 1.416 (95% CI 1.132–1.770) 7 days after treatment. On the other hand, only the fact of being the primary teeth used for mastication due to the absence of the contralateral tooth (*p* = 0.018) significantly affected PP occurrence 15 days after treatment with an OR = 18.4 (95% CI 1.6–207.2).

### 3.3. Intensity of PP

In terms of intensity of pain, Table 2 shows the results (n) at the different time intervals. The extent of apical enlargement and the presence of procedural errors did not significantly influence PP intensity; however, the ordinal regression analysis showed that both the arch where tooth is located (*p* = 0.04) and pulp vitality status (*p* = 0.004) significantly influenced intensity of PP 24 h after root canal treatment. More specifically, when present, PP was more intense in non-vital and mandibular teeth with an OR = 4 (95% CI 0.3–7.7) and OR = 8.5 (95% CI 2.7–14.3), respectively. The rest of registered factors did not have an influence either on the incidence or the intensity of PP at any other time interval.

## 4. Discussion

This prospective clinical study was designed with two purposes. On one hand, several studies have identified those patient and tooth-related factors that play a role in the incidence of PP and they were confirmed in the present study [1,2,4,6]; on the other hand, this study tried to address the lack of information available in scientific literature regarding how some intraoperative decisions and procedural errors that occur during treatment could influence PP occurrence.

Patients referred to the department for initial root canal treatment were included in this study. The perception of pain is extremely subjective and dependent on the cultural, individual and economic background of patients. This is an important limitation for this type of studies. To minimize this limitation and considering the idiosyncrasy of patients seeking for treatment in university settings, a trained assistant was in charge of assessing the incidence and intensity of PP through a telephone interview in the first 24 and 48 h and patients were required to attend the facility for personal interviews 7 and 15 days after treatment. These specific time points were selected to understand the role of the different factors related to immediate PP (24 and 48 h) first; and furthermore, those that contributed to the maintenance of a prolonged duration of PP (7 and 15 days after treatment). Moreover, different scales have been described along endodontic literature for the subjective measurement of PP. For further simplicity and as recommended in a Cochrane Review [3], the incidence and intensity of pain was inquired based on a simple verbal categorization defined by the need for and relief from analgesic intake in the present study.

The global incidence of PP detected in the present study (29.4%) is in accordance with those reported by earlier reports [11]. However, other studies have shown much higher rates of PP [1,6]. The difference with these studies may be explained by variations in treatment protocols and inclusion criteria; but most importantly, all participants in the present study were asked to take postoperative analgesics immediately after root canal treatment, whereas the criteria for postoperative analgesic intake is not rigidly established in other studies [1,6]. The reason for advising analgesics intake immediately after treatment in the present study is based on recommendations from earlier reports [16,17]. The immediate administration of analgesics has demonstrated effective pain relief and a reduction in the need for further analgesic intake [16,17,18,19]. This also may be the reason for the low rate of PP in patients presenting preoperative pain. Posttreatment analgesics were able to control pain in 58.2% patients with immediate preoperative pain. Only five patients presented with unscheduled intervention or “flare-ups” out of the total 435 patients and this fact could not be related to any specific factor.

No clear evidence was available in the literature regarding the influence of apical size enlargement and postoperative pain occurrence. In the present study, root canal preparation was performed with NiTi rotary instruments up to size #25.06. Further apical enlargement was performed with hand instruments, but with care not to push apical debris beyond the root canal terminus. There is still controversy in the literature regarding the appropriate apical enlargement for disinfection purposes [20]; but, both the findings of the present study and those from a previous report [21] agree that larger preparations may influence PP appearance or duration. In fact, the final apical preparation size significantly influenced the incidence of PP seven days after treatment (62.5% out of the 3.7% of patients that still suffered PP seven days after treatment were prepared up to a minimum #40 final apical size).

The only factors that significantly influenced PP 24 and 48 h after treatment in the present study were the presence of immediate preoperative pain and the need for additional intraoperative anesthesia. The finding of immediate preoperative pain within 24 h having significant influence on postoperative pain occurrence for 24 h and 48 h is in agreement with earlier reports [1,2,6]. Hyperalgesia and allodynia occurring by both peripheral and central mechanisms in preoperatively symptomatic tooth has been reported to persist even after the dental treatment has been completed [22]. Thus, patients with increased preoperative pain have a higher risk of experiencing postoperative pain [22]. In fact, preoperative pain had been repeatedly cited in the literature as the most significant predictor for PP occurrence. Another report justifies the increased PP occurrence in symptomatic teeth suggesting that any possible pre-existing inflammation could worsen with the treatment and that patients with preoperative pain tend to expect and report higher rates of PP [23]. “Hot tooth” or occurrence of intra- operative pain within root canal treatment procedures has been associated with a less favorable post treatment result due to the increased anxiety experience and reduced pain threshold for patients with inadequate anesthetic effect [24]. The present study showed that if the patient required additional intraoperative anesthesia the odds ratio for experiencing PP increased 4.8 and 5.8-fold, respectively 24 and 48 h after treatment. Moreover, immediate preoperative pain still played an important role seven days after treatment.

At the same time, the only factor that significantly affected PP occurrence 15 days after treatment was the fact of being the primary teeth used for mastication due to the absence of the contralateral tooth. This parameter showed the highest OR when compared to any other parameter for all time intervals tested. Interestingly, the absence of the contralateral tooth increased the odds of suffering PP after 15 days by almost 20-fold (OR = 18.4 (95% CI 1.6–207.2)). The evidence of occlusal contacts inducing postoperative pain following root canal treatment has been somehow controversial [25,26,27,28,29]. Some studies observed a higher incidence of PP when occlusal contacts were present [2,24,25,26], but Mehrvarzfar et al. could not find any association [27]. However, they only included cases with mild or no preoperative pain and those teeth defined as having excess or greater than normal occlusal contacts were excluded from the study [27]. In the current investigation, the excessive load that a patient may exert when the contralateral tooth is not present may be responsible for a prolonged PP possibly for a delayed or compromised periapical inflammatory resolution.

Other variables registered in the present study like age, gender or the presence of periapical pathology did not show any significant association with PP occurrence. In accordance to these results, it has been reported a very small impact of the age of patients on the incidence of PP [13]; however, there is no agreement in the literature about the role of gender on PP occurrence following root canal treatment with certain observations concluding that gender has no influence [30], whereas others reporting that females have an increased propensity to suffer PP [1,13]. The subjective component of the pain experience may be the reason the variable “gender” varies among the different studies. The situation for women differs among countries and this fact itself may explain the lack of agreement among studies. In contrast to the results in the present study, Ng et al. reported a significant association between the size of periapical lesion and PP occurrence [1], although the sample size of teeth presenting periapical lesions determined by Ørstavik’s PAI score was very reduced (6.8%) in the present report. Finally, the present study showed that procedural errors did not influence the incidence of PP at any time interval after treatment. Future studies should address if long-term outcome of treatments is affected by this parameter.

In conclusion, this study confirmed the role of immediate preoperative pain in the incidence of post-endodontic pain 24 h, 48 h and 7 days after treatment and identified some new factors that worsen the chances of suffering PP. Specifically, the need of intraoperative anesthesia was associated with higher incidence of PP 24 and 48 h after treatment; the extent of apical enlargement played a significant role in the presence of PP after seven days of treatment; and the excessive occlusal load induced by the absence of a contralateral tooth was the only factor related to the maintenance of PP up to 15 days.

## Figures and Tables

**Table 1:** Incidence of postoperative pain and data distribution for relevant factors at the different time intervals (n). Total sample size was 435.

			24 h		48 h		7 Days		15 Days	
			Yes	No	Yes	No	Yes	No	Yes	No
Gender		Male	48	131	25	154	3	176	2	177
		Female	71	185	50	206	13	243	1	255
Group of teeth	Anterior	Max.	18	72	10	80	2	88	0	0
		Mand.	2	16	2	16	1	17	2	0
	Premolar	Max.	16	48	9	55	3	61	0	1
		Mand.	12	31	10	33	5	38	90	18
	Molar	Max.	23	48	8	63	1	70	62	43
		Mand.	48	101	36	113	4	145	71	148
Additional anesthesia		Yes	31	20	24	27	2	49	0	51
		No	86	291	50	327	14	363	3	374
Immediate preoperative pain		Yes	70	106	51	125	13	163	2	174
		No	48	210	24	234	3	255	1	257
Pulpal vitality		Yes	100	225	62	263	12	313	2	323
		No	19	91	13	97	4	106	1	109
Periapical radiolucency		Yes	7	23	5	25	2	28	0	30
		No	112	292	70	334	14	390	3	401
Presence of contralateral tooth		No	15	28	9	34	3	40	2	41
		Yes	104	288	66	326	13	379	1	391
Procedural errors		Yes	15	36	10	41	4	47	1	50
		No	104	280	65	319	12	372	2	382
Apical enlargement		6% taper #20	8	28	2	31	1	32	2	31
		6% taper #25	45	184	54	214	5	263	1	267
		#35	0	8	0	8	0	8	0	8
		#40	18	45	13	50	7	56	0	63
		#45	2	19	2	19	1	20	0	21
		More than #45	10	31	4	37	2	39	0	41

**Table 2:** Results (n) for general incidence and intensity of pain. Total sample size was 435.

Incidence	Intensity	Time Interval			
		24 h	48 h	7 Days	15 Days
NO		314	361	419	432
YES	Mild	85	59	14	3
	Moderate	28	10	2	0
	Intense	8	5	0	0
